# Supplementary material for: Unraveling the Phase Transition Behavior of MgMn2O4 Electrodes for Their Use in Rechargeable Magnesium Batteries
Source: Materials (Basel). 2023 Aug 1;16(15):5402. doi: 10.3390/ma16155402 (PMC10419908; doi:10.3390/ma16155402)
Supplement: Supplementary file 1 [file materials-16-05402-s001.zip › materials-2455173-supplementary.pdf]

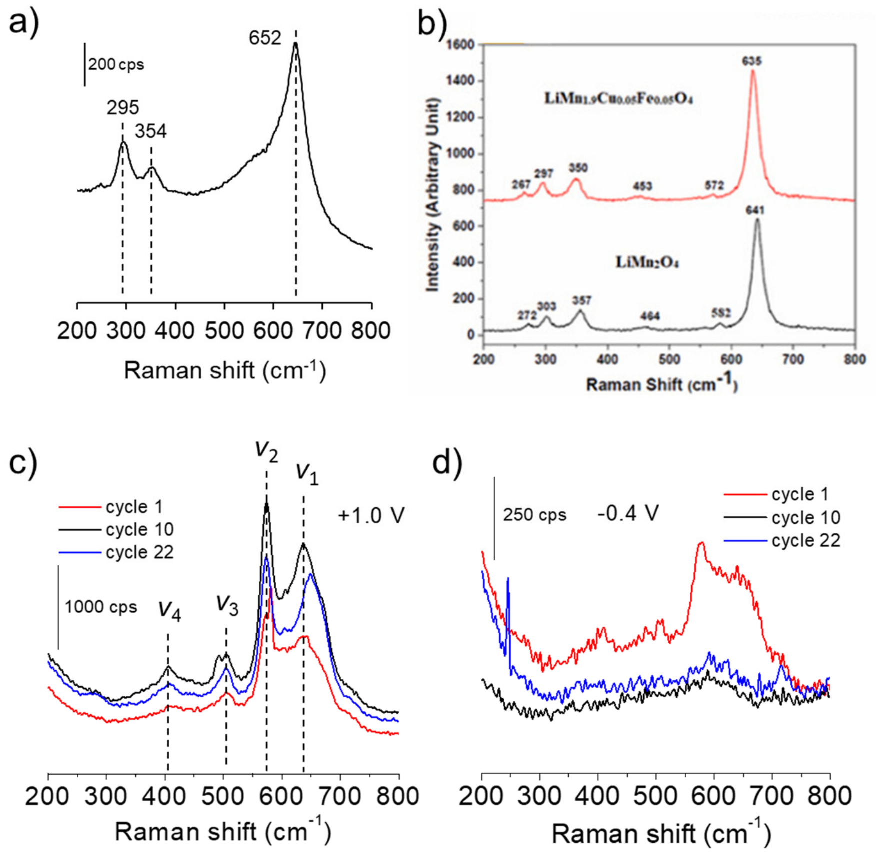

Figure S1. a) Raman spectrum for the as-prepared $MgMn_2O_4$ powder; b) Raman spectrum reported for $LiMn_2O_4$ and $LiMn_{1.9}Cu_{0.05}Fe_{0.05}O_4$ reproduced with permision from *Results in Materials*, vol. 21, Taddesse, P.; Gebrekiros, H.; Semu, G.; Duressa, M.; Chemeda, Y.C.; Murali, N.; Babu, K.V. Investigation of Structural, Vibrational Spectroscopic and Properties Study of $LiMn_2O_4$ and $LiMn_{1.9}Cu_{0.05}Fe_{0.05}O_4$ Cathode Materials, 100224, Copyright Elsevier (2021); c) and d) Raman spectrum evolution with the number of voltammetric cycles for a $MgMn_2O_4$ electrode in 1.0 M $Mg(NO_3)_2$ at 1.0 V (oxidized, charged) and -0.4 (reduced, discharged), respectively. Potentials are referred to a Ag/AgCl/KCl (3 M) electrode.

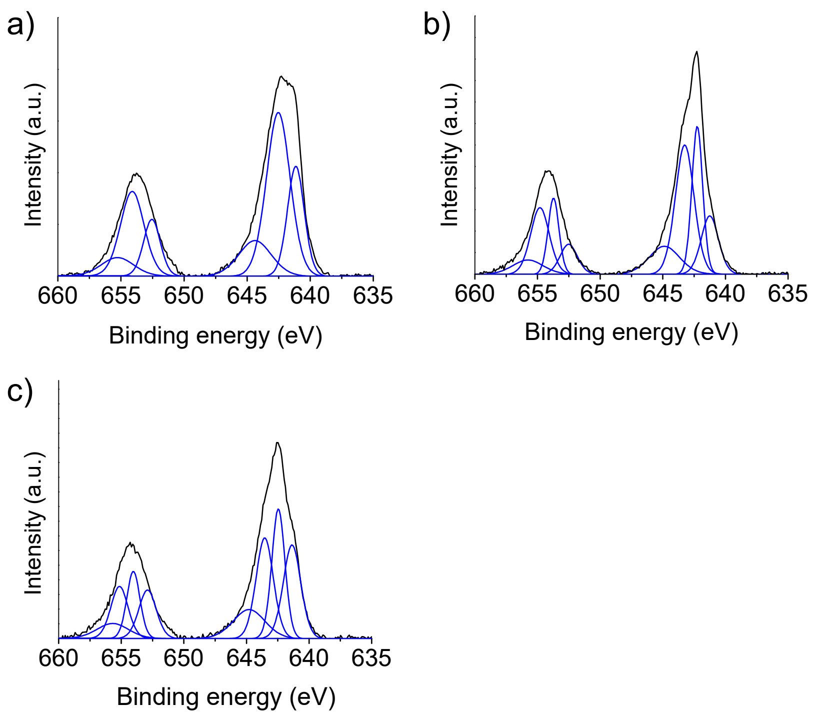

Figure S2. XPS deconvolution analysis of Mn 2p of $MgMn_2O_4$ for different states of charge. a) $MgMn_2O_4$ freshly prepared. b) Oxidized at 1.0 V and c) reduced at -0.7 V in 1.0 M $Mg(NO_3)_2$ after 15 successive cycles at 5 mV s^{-1}.

Table S1. XPS Mn $2p_{3/2}$ spectral fitting parameters: binding energy (eV), area of each contribution and percentage of total area.

		Mn(III)		Mn(IV)	
$Mg_{0.8}Mn_2O_4$	Mn 2p peak (eV)	641.14	642.53	644.38	646.53
	Area (arb. units)	69.24	145.57	43.95	0
	Total area for each species (arb. units)	214.81		43.95	
	Total area (arb. units)	258.76			
	Area (%)	83.02		16.98	
Oxidized spinel	Mn 2p peak (eV)	641.25	642.25	643.25	644.88
	Area (arb. units)	45.78	72.69	109.52	38.89
	Total area for each species (arb. units)	118.47		148.41	
	Total area (arb. units)	266.88			
	Area (%)	44.39		55.61	
Reduced spinel	Mn 2p peak (eV)	641.36	642.45	643.54	644.79
	Area (arb. units)	54.82	56.61	58.16	30.99
	Total area for each species (arb. units)	111.43		89.15	
	Total area (arb. units)	200.58			
	Area (%)	55.56		44.44	

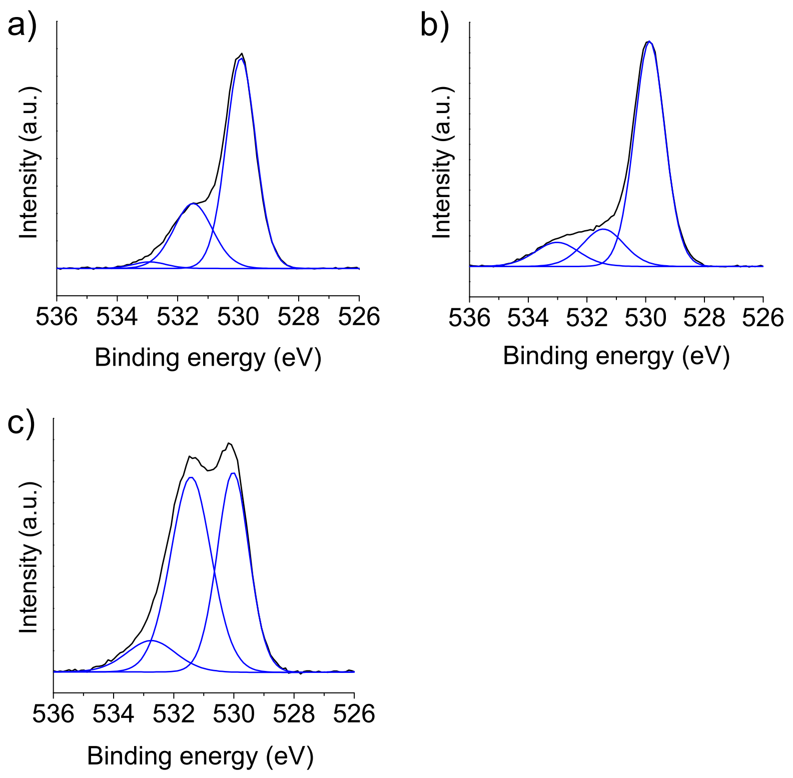

Figure S3. XPS deconvolution analysis of O 1s for $MgMn_2O_4$ in different states of charge. a) $MgMn_2O_4$ freshly prepared. b) Oxidized at 1.0 V and c) reduced at -0.7 V in 1.0 M $Mg(NO_3)_2$ after 15 successive cycles at 5 mV s^{-1}.

Table S2. XPS O 1s spectral fitting parameters: binding energy (eV), area of each contribution and percentage of total area.

		Mn-O-Mn	Mn-OH	H-O-H
$Mg_{0.8}Mn_2O_4$	O 1s peak (eV)	529.91	531.49	532.93
	Area (arb. units)	532.72	215.85	18.52
	Total area (arb. units)	767.09		
	Area (%)	69.45	28.14	2.41
Oxidized spinel	O 1s peak (eV)	529.87	531.44	533.02
	Area (arb. units)	544.57	121.92	84.38
	Total area (arb. units)	750.87		
	Area (%)	72.52	16.24	11.24
Reduced spinel	O 1s peak (eV)	530.03	531.43	532.75
	Area (arb. units)	357.46	442.86	86.17
	Total area (arb. units)	886.49		
	Area (%)	40.32	49.96	9.72

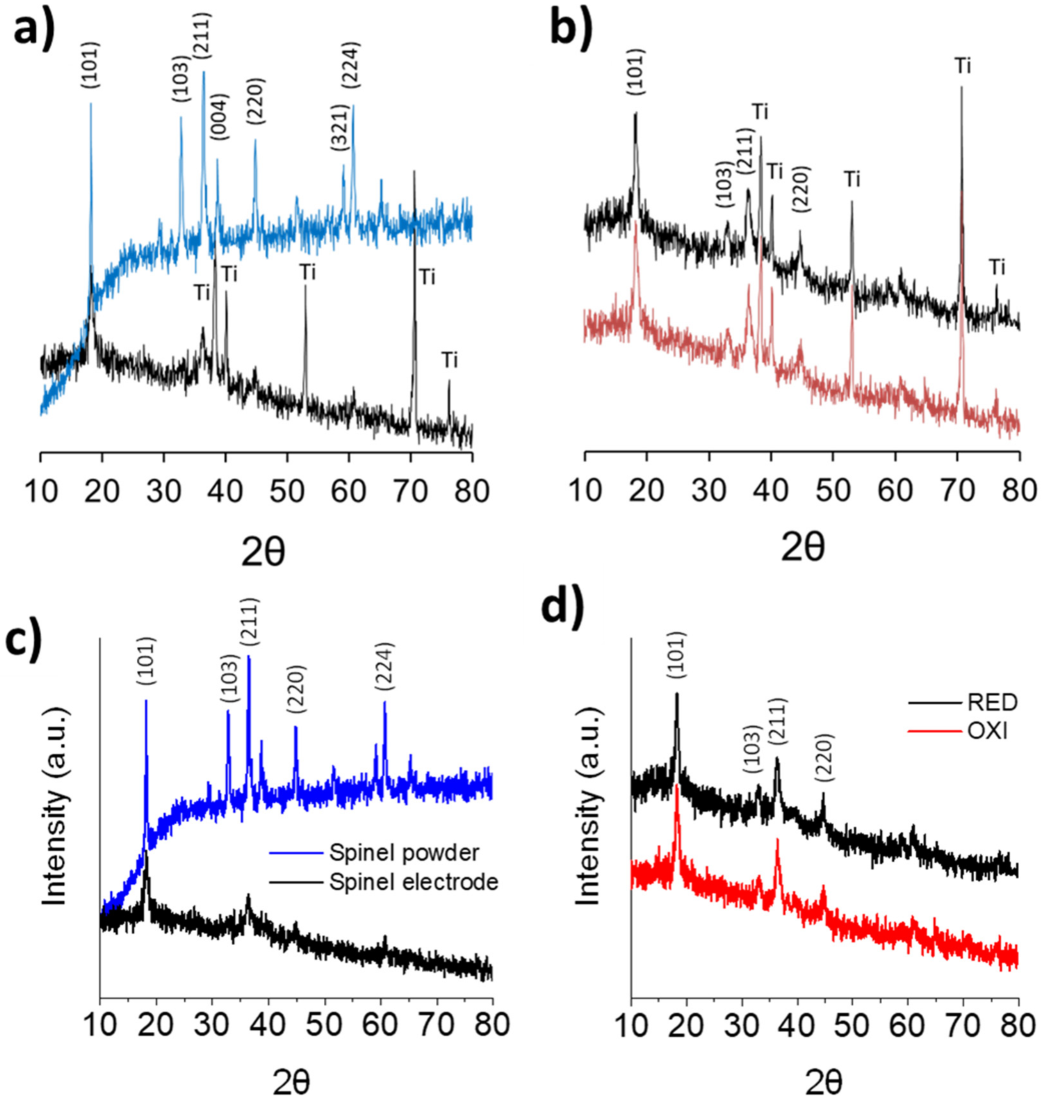

Figure S4. a) XRD for freshly prepared $MgMn_2O_4$ powder (blue) and for an electrode after 15 voltammetric cycles (black). b) XRD for a reduced $MgMnO_4$ electrode at -0.7 V (black) and for an oxidized electrode at 1.0 V (red) after 15 cycles (in 1.0 M $Mg(NO_3)_2$ aqueous solution at 5 mV s^{-1} and using titanium foil as a substrate. The JCPDS Card no. 00–044–1294 was employed to identify the peaks corresponding to the Ti substrate (indicated in the Figure) while the JCPDS Card no. 01-072-1336 was employed to identify tetragonal $MgMnO_4$. For clarity, Figures c) and d) show the XRD patterns after subtracting the Ti substrate contribution.

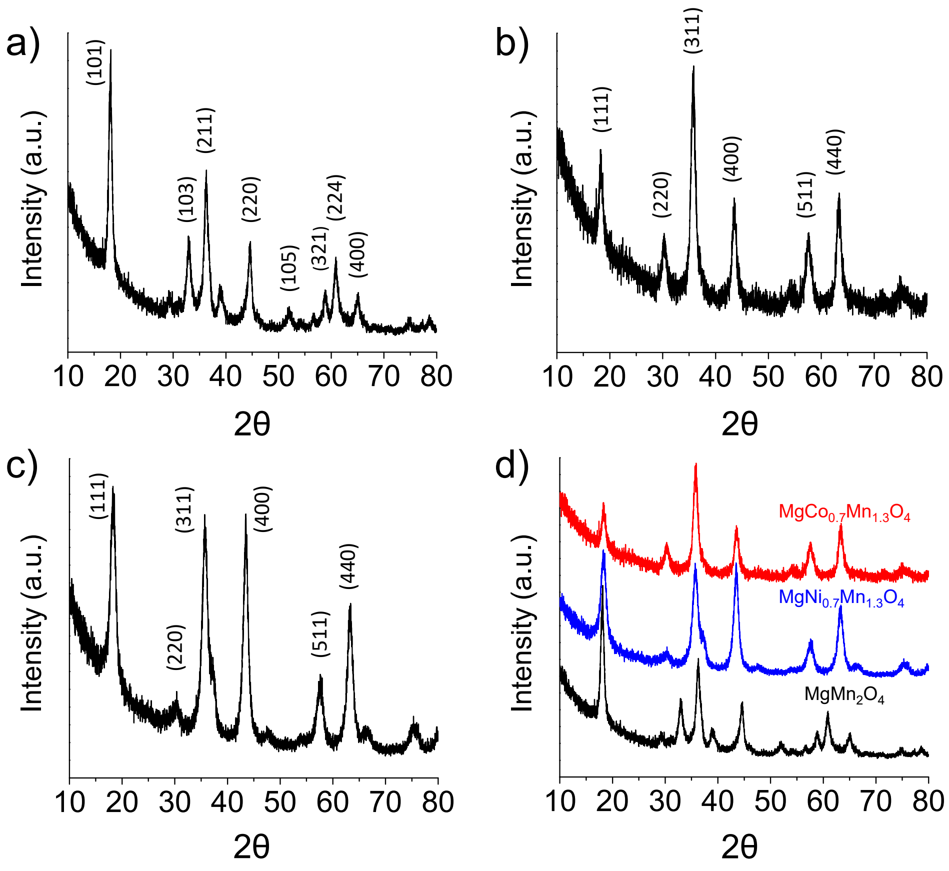

Figure S5. XRD patterns for powders of $MgMn_2O_4$ (black) (a), $MgCo_{0.7}Mn_{1.3}O_4$ (red) (b), and $MgNi_{0.7}Mn_{1.3}O_4$ (blue) (c). For the sake of comparison, panel (d) shows the three patterns.

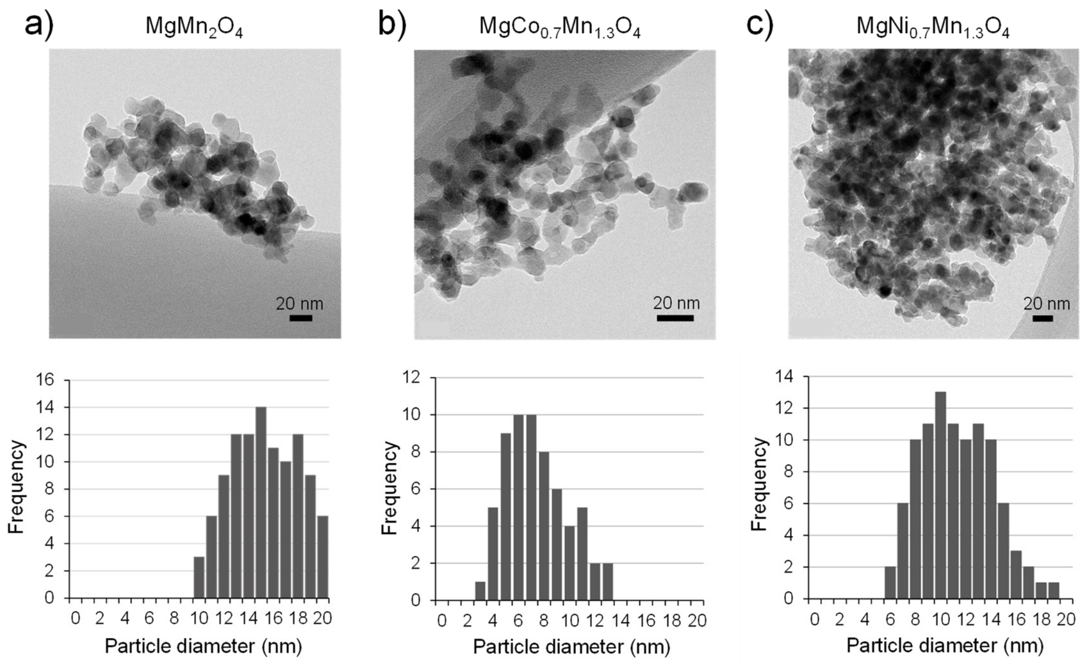

Figure S6. TEM images and the corresponding histograms for pristine $MgMn_2O_4$ (a), $MgCo_{0.7}Mn_{1.3}O_4$ (b), and $MgNi_{0.7}Mn_{1.3}O_4$ (c) powders.

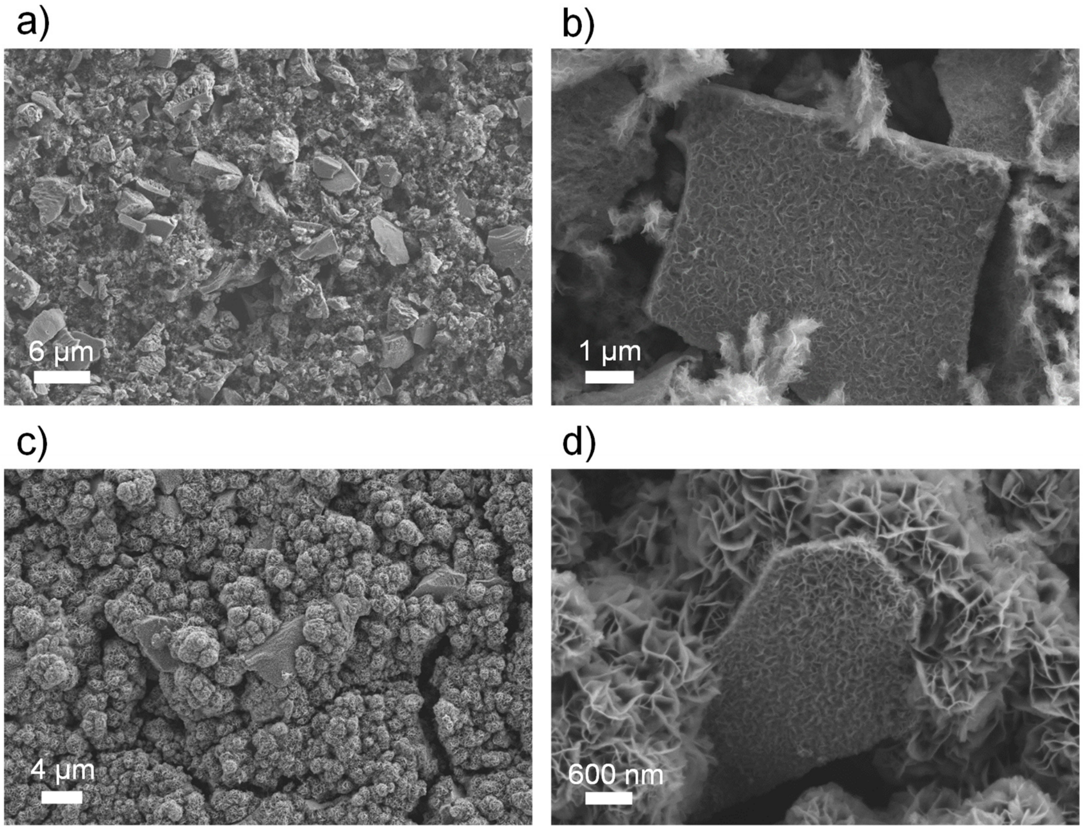

Figure S7. FE-SEM images for $MgCo_{0.7}Mn_{1.3}O_4$ electrodes: (a) freshly prepared, (b) and (c) after 10 voltammetric cycles, and (d) after 120 cycles. Scan rate: 5 mV s^{-1}; Electrolyte: 1.0 M $Mg(NO_3)_2$.
